# Review of the Online One Welfare Portal: Shared Curriculum Resources for Veterinary Undergraduate Learning and Teaching in Animal Welfare and Ethics

**DOI:** 10.3390/ani10081341

**Published:** 2020-08-03

**Authors:** Paul D. McGreevy, Anne Fawcett, Jane Johnson, Rafael Freire, Teresa Collins, Chris Degeling, Andrew D. Fisher, Susan J. Hazel, Jennifer Hood, Janice K. F. Lloyd, Clive J. C. Phillips, Kevin Stafford, Michelle L. Hyde, Bethany Wilson, Vicky Tzioumis

**Affiliations:** 1 Sydney School of Veterinary Science, University of Sydney, Sydney, NSW 2006, Australia; anne.fawcett@sydney.edu.au (A.F.); michelle.hyde@sydney.edu.au (M.L.H.); bethany.wilson@sydney.edu.au (B.W.); Vicky.tzioumis@sydney.edu.au (V.T.); 2 Department of Philosophy, Hearing Hub, Macquarie University, North Ryde, NSW 2109, Australia; jane.johnson@mq.edu.au; 3 School of Animal and Veterinary Science, Institute for Land, Water and Society, Charles Sturt University, Wagga Wagga, NSW 2650, Australia; rfreire@csu.edu.au; 4 School of Veterinary Medicine, College of Science, Health, Engineering and Education, Murdoch University, Murdoch, WA 6150, Australia; t.collins@murdoch.edu.au (T.C.); jenih@iinet.net.au (J.H.); 5 Australian Centre for Health Engagement, Evidence and Values, University of Wollongong, Wollongong, NSW 2522, Australia; degeling@uow.edu.au; 6 Faculty of Veterinary Science, University of Melbourne, Parkville, VIC 3100, Australia; adfisher@unimelb.edu.au; 7 School of Animal and Veterinary Sciences, University of Adelaide, Roseworthy, SA 5005, Australia; susan.hazel@adelaide.edu.au; 8 College of Public Health, Medical and Veterinary Sciences, James Cook University, Townsville, QLD 4811, Australia; janice.lloyd@jcu.edu.au; 9 School of Veterinary Science, University of Queensland, Gatton, QLD 4343, Australia; c.phillips@uq.edu.au; 10 Institute of Veterinary, Animal and Biomedical Sciences, Massey University, Palmerston North 4442, New Zealand; k.j.stafford@massey.ac.nz

**Keywords:** animal ethics, animal welfare, veterinary medicine, online curriculum resources, learning and teaching, One Welfare

## Abstract

**Simple Summary:**

All of the veterinary schools in Australia and New Zealand worked together to develop the online One Welfare learning and teaching portal (OWP) for sharing teaching resources to assist veterinary graduates to become leaders in animal welfare and ethics (AWE). The materials in the portal are organised around eight key themes including two overarching themes of animal ethics and animal welfare science, and six context-specific themes: companion animals; animals used in research and teaching; livestock/production animals; animals used for sport, recreation or display; animals in the wild, and aquatic animals. The arrangement of these resources aligns with those of the Australian Animal Welfare Strategy (AAWS). As part of the OWP development, animal welfare educators from the eight veterinary schools met and used modified deliberative polling to prioritise resource development. Surveys of students and educators in all participating schools investigated their attitudes to current AWE issues across six context-specific themes: companion animals; animals used in research and teaching; livestock/production animals; animals used for sport, recreation or display; animals in the wild and aquatic animals. The prioritised resources include (1) student AWE essays arranged by subject; (2) an online reflection tool that can be used repeatedly to gain understanding of change in attitudes over time; (3) eight overarching themes that host a group of interactive scenarios; (4) research papers for each context-specific theme; (5) a bank of multiple choice questions with feedback to support AWE assessment; (6) a novel online discussion tool ‘Chatterbox;’ and (7) a ‘toolbox’ containing directions to the doglogbook, an owner app designed to monitor management practices, and evaluate quality of life in dogs.

**Abstract:**

This article introduces the online One Welfare learning and teaching portal (OWP) and describes its development, use, importance and relevance to animal welfare and ethics (AWE) stakeholders. As animal welfare issues increase in importance, veterinarians must be trained to lead the science that underpins AWE discourses. The OWP is a collection of resources designed to engage and challenge veterinary science students as they become advocates for animals. It was developed collaboratively by all eight veterinary schools in Australia and New Zealand, and funded by the Australian Government Office for Learning and Teaching. Surveys to investigate the attitudes of students and educators to AWE issues in six context-specific themes based on the Australian Animal Welfare Strategy (AAWS) (companion animals; animals used in research and teaching; livestock/production animals; animals used for sport, recreation or display; animals in the wild and aquatic animals) were administered through all participating schools. Students assigned more importance to Day One competence in knowledge of welfare concepts than did educators for the following groups: production animals, companion animals, animals in the wild, aquatic animals, animals used in research and teaching, and animals used for sport, recreation or display (all *p* < 0.01). Agreement between educators and students was closer regarding the importance of Day One competence for euthanasia for all six context-specific themes (*p* < 0.01–0.03). Students assigned more importance than educators to *social, economic and cultural drivers* of welfare outcomes in production animals (*p* < 0.01); *slaughter and preslaughter inspections* in production animals (*p* < 0.01); *animal abuse and hoarding* in companion animals (*p* < 0.01); *shelter medicine* in companion animals (*p* < 0.01); *disaster preparedness* in wildlife animals (*p* < 0.01); *pain and distress caused by fishing* in aquatic animals (*p* < 0.01); *conscientious objection* related to animals held for research and teaching (*p* < 0.01); *behaviour, selection and training* of animals used for sport, recreation and display (*p* = 0.046) and *educating the public* around sporting animal welfare (*p* < 0.01). Agreement between educators and students was closer for *strategies to address painful husbandry* *procedures* in production animals (*p* = 0.03); *behaviour and training* of companion animals (*p* = 0.03); *veterinarians’ duties to wild animals* in wildlife (*p* = 0.02); *the 3Rs* in animals held for research and teaching (*p* = 0.03) and *ownership responsibility* in sporting animals (*p* = 0.01). This report discusses the reasons for differences among students and educators as they approach these issues. The portal is expected to gather more content as veterinary schools in other countries use its resources and users submit scenarios and discussion topics that reflect local needs.

## 1. Introduction

A compelling argument exists that animal welfare should be at the core of veterinary education. The World Organization for Animal Health (OIE) stated in 2012 that veterinarians should be “the leading advocates for all animals, recognising the key contribution that animals make to human society through food production, companionship, biomedical research and education” [1]. In the same year, the British Veterinary Association (BVA) acknowledged the need for veterinarians to advocate for animals [2] and in 2016 the Australian Veterinary Association (AVA) placed ‘improving animal welfare’ as its first strategic priority [3]. In 2017, the OIE published its Global Animal Welfare Strategy with a vision of “a world where the welfare of animals is respected, promoted and advanced, in ways that complement the pursuit of animal health, human wellbeing, socioeconomic development and environmental sustainability” [4]. This is consistent with a ‘One Welfare’ approach [5]. One Welfare describes an approach to welfare that mirrors the way One Health connects animal health to human health and the health of the environment. One Welfare recognises the inextricable links between animal welfare, human welfare and societal mental health, as well as environmental conservation and sustainability [6,7].

Globally, there are increased community concerns about food security and ethical food production [8] and animal welfare [9]. Indeed, the 2018 Ketchum Poll, an annual omnibus online survey of 1000 Americans, identified animal welfare as the number one issue of concern among a sample of US adults [10].

The growing awareness of the One Welfare approach [5,11] may make animal welfare easier for stakeholders to discuss simply because it asks for some assessment of how animal welfare can be improved along with human welfare and planetary wellbeing. Veterinarians are ideally placed to be AWE leaders due to their contact with clients, patients, animal industries, colleagues and the community. While students undoubtedly enter veterinary school with their own experiences, understandings and viewpoints, the veterinary curriculum is a key source of their formal AWE education. Students are also influenced by the ‘hidden curriculum,’ which describes the usually unacknowledged and unspoken things they acquire outside the formal curriculum, as well as by the opinions and behaviours of their instructors and peers [12]. This informal learning can be positive or can conflict with the values, behaviours and norms of the formal curriculum. Neve and Collett (2018) assert the hidden curriculum can have a powerful influence on medical students’ professional development [13]. Veterinarians have a range of ethical obligations to a variety of stakeholders, as well as the personal overall responsibilities they share with the ‘general public’ [14]. In 2013, Yeates [15] highlighted the pivotal ethical position of veterinarians as having “greater accountability because of more opportunities to cause greater harms,” and “fewer excuses” due to their greater knowledge.

As well as companion and production animals, veterinarians may also care for animals in the contexts of sport, display and recreation, wildlife, research and teaching. All use of animals for teaching and research purposes in Australia and New Zealand comes with legal and moral responsibilities. Central to these responsibilities is the need to abide by the 3Rs (Replacement, Reduction and Refinement) of animal use in teaching and research [16]. The same principles can be applied to the use of animal welfare science in ethical decision-making.

As part of the development of the online One Welfare teaching portal, animal welfare educators from the eight Australian and New Zealand veterinary schools (hereafter referred to as the panel) met in April 2014 for a two-day workshop at the University of Sydney and used modified deliberative polling to prioritise resource development, described in detail in Johnson et al. 2015 [17]. The purpose of this workshop was to harness the collective expertise of the panel to develop online teaching resources via a shared understanding of the key AWE competences required of veterinary graduates. In summary, the panel divided AWE topics into six themes that align with those of the Australian Animal Welfare Strategy (AAWS) [18] and two broad overarching areas, which became the key subject areas. A number of reports arose from this collaboration, namely, Degeling et al. 2017 [19], Collins et al. 2018 [20], Cornish et al. 2018 [21], Fawcett et al. 2019 [22], Stafford et al. 2019 [23], Lloyd et al. 2020 [24], Cornish et al. 2016 [25] and Freire et al. 2016 [26]. Broadly, these have described how veterinary undergraduate students prioritise the importance of AWE topics as so-called Day One competences (see below) for new graduates. Additionally, veterinary science educators in Australia and New Zealand were surveyed on their opinions of the importance of these competences [27].

The aim of the current article is to summarise the findings of the research that underpinned the development of the OWP, to introduce the resources it hosts and to present the reasons for developing the resources.

## 2. Materials and Methods

### 2.1. The Panel’s Prioritisation of Online Resource Development for the OWP

On the first day of the 2014 workshop, panel members who represented AWE teachers from each school were given information on a selection of teaching tools and learning activities (Table 1), which they discussed in detail. They then ranked AWE topics on a scale of one (*extremely important*) to 10 (*least important*); according to their perceived importance as Day One competences, as identified by the UK Royal College of Veterinary Surgeons (RCVS) [27]. Day One competences refer to the important principles—knowledge, skills, attitudes and behaviours— that all students must achieve before they graduate, so that they are safe to practice in all veterinary contexts from day one after graduation [28].

### 2.2. Educators’ Perspectives on Key AWE Day One Competences Required of Veterinary Graduates

Educators from the eight Australian and New Zealand universities teaching veterinary science were invited to participate in a survey (constructed using SurveyMonkey [29]) asking them to indicate their opinions on the importance of various AWE topics on graduates’ Day One competences, as well as stipulating their own teaching focus. The survey comprised 11 questions with three demographic questions; a question asking about the subjects the respondents taught; and then seven questions assigned to relevant sections of veterinary employment related to Day One competences of Australian and New Zealand graduates. Seven employment sections were used: general practice; production animals; companion animals; wild animals; aquatic animals; animals kept for scientific purposes; and animals used in sport, recreation and display (see Table 2). Each of these sections further referred to AWE topics relevant to each area of clinical veterinary practice. A 10–point Likert scale (*ranging from 1 = extremely important to 10 = least important*) allowed respondents to indicate their importance of each topic for graduates as a Day One competence.

### 2.3. Students’ Perspectives on Day One Competences in AWE

Students enrolled in veterinary science or veterinary medicine programs at Australian and New Zealand universities during October 2014 were invited to participate in an online survey administered using SurveyMonkey [29]. The survey was developed by the panel. Voluntary participation of students was sought via three emails sent a week apart. All participating institutions were guaranteed anonymity and each institution provided human ethics committee approval. The questionnaire was quantitative in nature and comprised 12 questions.

Students in years 1 and 2 were classed as early students, years 3 and 4 as mid–stage students, and years 5 and 6 as senior students. Students were asked: (1) to provide information on the importance of selected AWE topics as Day One competences by using a 10–point Likert scale (ranging from *1 = extremely important to 10 = least important*); (2) about their career preferences and opinions with respect to the perceived importance of various topics in animal welfare on Day One of practice and (3) what they consider to be important Day One competences in the seven identified areas of veterinary practice (Table 2).

Difference in the Importance scores assigned by students and educators were analysed using the lm function within the R statistical software. Median values were used due to the ranked nature of the ordered dataset obtained.

## 3. Results

### 3.1. Prioritisation of Development of Online Resources by Educators

The panel decided that the goals of the AAWS should be the focus of deliberations. These are: a national approach to animal welfare; sustainable improvements to animal welfare; and improved communication, education and training.

As all Australian and New Zealand veterinary schools currently have RCVS accreditation, it was also agreed that RCVS Day One competences relating to AWE needed to be addressed.

The panel ranked the methods and resources listed in Table 1 in terms of importance (where 1 = most important and 10 = least important) for preparing students for Day One competences (based on those of the RCVS) as shown in Table 3.

The educators ranked the ‘scenarios and learning exercises’ highest, followed by the ‘QOL assessment tool,’ and then ‘a negotiated curriculum’ and the ‘human continuum’ and in equal third ranking (Table 3).

### 3.2. Educators’ Perspectives on Key Animal Welfare and Ethics Competences Required of Veterinary Graduates

The survey of educators had a response rate of 25% (142 participants of 550 surveyed) of whom 51% were female. The results are summarised in Table 4.

### 3.3. Students’ Perspectives

All students enrolled in veterinary science courses or veterinary medicine programs in Australia and New Zealand were invited to participate in the online survey. Of the 3220 veterinary students enrolled in Australia and New Zealand in October 2014, 851 participated, resulting in an average response rate of approximately 25%. Of these, 671 were female (79%), 145 male (17%), and 35 people (4%) did not complete the gender item.

Log linear modelling was used to analyse the three–way contingency tables of frequencies associated with (1) gender and (2) stage of study. Given that there were fewer than 20 males in the senior years of studies (across all universities) scores of six or more for the two statements were combined for analysis [26].

Students ranked *triage* (median = 3) and *professional ethics* (median = 4) as the two most important Day One AWE competences. *Perspectives on welfare* (median = 8) and *understanding the development of animal welfare science* (median = 9) were ranked as the least important. Senior students ranked *triage* as more important than mid and early stage students. Senior students also ranked *having an understanding of the reason why animal welfare matters* less important than mid and early stage students. *Professional ethics* was also ranked as more important by early and mid–stage students. Female students ranked the *importance of the animal-human* bond more highly than male students, but also recorded a reduction in importance of the topic of *understanding of perspectives on welfare* as a Day One competence over the course of the degree.

#### 3.3.1. Career Preferences and Opinions on Animal Welfare and Ethics

A subset of the survey questions explored students’ career preferences and attitudes to animal welfare. Most students expressed the desire to work in mixed practice (30.1%); followed by companion animal practice (25.2%); production animal practice (10%); exotic animals (9.4%); equine practice (7.6%); research (5.1%); and government work (5.4%). Less than five per cent (4.8%) of students said they did not know what sort of practice they wanted to work in after graduation.

#### 3.3.2. Welfare and Ethics Issues in Various Areas of Veterinary Practice

The last section of the questionnaire asked students what Day One competences they considered to be important in various areas of veterinary practice. Table 5 summarises the findings.

### 3.4. Differences Between Importance Rankings by Educators and by Students

In general, students ranked first day knowledge of AWE as more important than did educators for production animals (t = −10.401, *p* < 0.01), companion animals (t = −14.655, *p* < 0.01), wildlife (t = −10.721, *p* < 0.01), aquatic animals (t = −8.514, *p* < 0.01), scientific research animals (t = −11.782, *p* < 0.01) and animals used in sport recreation and display (overall t = –8.518, *p* < 0.01). For each of these topics, agreement was better between educators and students on the importance of first day competence about *euthanasia* (t values for interaction effects between 2.15−64.908, *p* < 0.01–0.03).

In addition, agreement between students and educators was closer for the importance of *strategies to address painful husbandry procedures* in production animals (t value for interaction effect = 2.154, *p* = 0.03); *behaviour and training* of companion animals (t = 2.2123, *p* = 0.03); *veterinarians’ duties to wild animals* in animals in the wild (t = 2.408, *p* = 0.02); *3Rs–Replacement, Reduction and Refinement* in animals used for scientific purposes (t = 2.172, *p* = 0.03); and *ownership/responsibility* in animals used in sport, recreation and display (t = 2.615, *p* = 0.01).

Conversely, there was significant disagreement between the Day One importance assigned by students and educators to *social, economic and cultural drivers of welfare outcomes* in production animals (t = −2.37, *p* < 0.01) and *slaughter and preslaughter inspections* in production animals (t = −2.851, *p* < 0.01); animal abuse/ hoarding in companion animals (t = −3.389, *p* < 0.01) and *shelter medicine* in companion animals (t = −3.763, *p* < 0.01); *disaster preparedness* (t = −2.853, *p* < 0.01) in wildlife animals; for *pain and distress* caused by pain and *distress associated with standard angling and trawling practices* in aquatic animals (t = −4.032, *p* < 0.01); for *conscientious objections* related to animals held for scientific purposes (t = −7.586, *p* < 0.01); and for *behaviour, selection and training for sport and recreation displays* (t = −1.999, *p* = 0.046) regarding sporting animals and for *educating the public* around animal welfare in animals used in sport, recreation and display (t = −2.800, *p* < 0.01).

## 4. Discussion

### 4.1. Prioritisation of Development of Online Resources by Educators

The use of scenarios for teaching AWE was supported by the panel, the members of which were all experienced in scenario-based animal welfare and ethics teaching. The use of scenarios is also common in human medical ethics teaching, which shares many of the issues and challenges of veterinary ethics teaching. The safe and supported learning environment provided by well-designed and expertly facilitated teaching scenarios acts to engage students in real life situations without the inherent danger of ‘making a mistake’ and harming animals or the people engaged with them.

The potential use of the QOL tool was considered by the educators to have merit on several levels, including stimulating students’ deep thinking about the complexities and challenges of QOL assessment, and their own roles in assessing animal QOL and making recommendations based on those assessments. The eventual tool, *doglogbook*, was also designed to help students to understand, and discuss with owners, issues such as animal QOL, end-of-life care, humane killing and euthanasia.

The OWP has had high quality students’ animal welfare science essays added annually since it launched in 2016. The skills of active listening, critical analysis and argument, as well as position development, are critical for success in advocacy and human behaviour change, and the human continuum exercise provides a safe and encouraging space for practising and developing these skills. The use of the online version of the human continuum allows students to take part in wide-ranging, robust discussions without face-to-face peer pressure [31].

A negotiated curriculum was seen by educators to be a core pillar of support for success of the anticipated online portal. One of the many challenges veterinary schools face is that of having to provide the requirements of formal tertiary qualifications, along with relevant, up-to-date and flexible curriculum content. Veterinary schools are also generally small in terms of financial and staffing resources, especially in comparison to medical schools. Great value could be made of targeted international collaboration to use the individual specialities of the collaborating veterinary schools to further increase relevance and diversity in curricula.

### 4.2. Educators’ Perspectives

Overall, the perspectives of veterinary educators should be carefully considered as they are likely to influence student attitudes. We revealed that veterinary educators rated *professional ethics*, *euthanasia* and *triage* as the three most important AWE topics for new graduates to have Day One competence. The topics ranked least important were *the development of animal welfare science* and *perspectives on welfare.* Except for general practice, educators considered that there were very important statements in all topics. Because AWE can appear in many disparate parts of the veterinary curriculum, it is worth acknowledging that educators’ areas of teaching focus can influence their views on AWE. Our studies have shown that educators teaching one or more ethics-related subjects were less likely to rate *neutering* and *euthanasia* as important as those not teaching these subjects. The reasons for this are currently unclear. However, these rankings are likely to shift as we see more debate about the complex nature of the decision to desex dogs [32] and the unintended harms from veterinary activities such as euthanasia [7]. It is also worth noting that veterinary educators may also be veterinary practitioners in any of the fields of practice and may have somewhat different viewpoints depending on their particular clinical focus (for example, a veterinary oncologist may encounter end-of-life decisions more often that a veterinary dermatologist).

In general, the results of the survey show that educators favour demonstrably practical issues, and this may represent a critical point if newly qualified veterinarians are to become more involved with AWE and advocacy. The responses of the educators were also in broad agreement with previous research that indicates that males are less concerned with animal welfare than females, and that females are more likely to show empathy towards some animals than do males [33]. That said, relatively few male staff members responded. 

### 4.3. Students’ Perspectives

Students were asked about AWE issues in various areas of veterinary practice and their importance among Day One competences. Their gender and stage of study were included in the analysis that has been reported in detail in Freire et al. (2016) [26] and in Table 5. In summary, at the beginning of their veterinary studies students considered *triage* the most important Day One competence and they also ranked an *understanding of triage* as increasingly important throughout the stages of their studies. *Professional ethics* was also identified as one of the most important Day One competences by early and mid–stage students, more than by those at the end of their studies. *An understanding of why animal welfare matters* declined in importance to students as they advanced through their studies and the more theoretical competences of the *development of animal welfare science* and *perspectives on animal welfare* were rated of low importance. It is interesting to consider that veterinary students may have some preoccupation with knowledge accumulation and practical skills that might be reflected in their high ranking of seemingly ‘practical’ topics among Day One competences.

Female students considered the *human-animal bond* as more important for Day One competence than did male students. Female students, but not males, showed a decline over the progression of their studies in importance for Day One competence in *an understanding of perspectives on welfare*. Given the small number of male participants in these samples, caution is needed when interpreting [25] these findings. Veterinary science had its origins in agricultural practice and was traditionally male dominated. However, in recent years, females have strongly dominated the student cohorts and approximately 80% of recent graduates in Australia are now female [34].

Stage of study also influences career preferences as does the cultural and educational milieu of the institution attended by the students. As they progressed through their studies, students showed an increasing preference for companion animal practice and a decreasing preference for production animal practice. This may reflect concern about working conditions, including after-hours and emergency work, salary, practice atmosphere, and compatibility with family duties in companion animal versus rural practice [35], and an increased focus on wellbeing in the curriculum, stressing the importance of work-life balance and self-care [36]. No effect of gender on preference for companion versus production animal practice was seen. Female students did consider *animal welfare* topics as being more important for new graduates than males throughout all stages of their study. As an indication of the many factors that influence Australian and New Zealand veterinary students’ career preferences, both males and females assigned less importance to *animal welfare* topics as Day One competences as they advanced through their studies. These results are reported in detail in Cornish et al. 2016 [25] and they align with other studies that have shown concern for animals declining with progression though veterinary degrees [37,38,39,40].

## 5. Conclusions

It is essential that new graduates are prepared to deal with the responsibilities of being a veterinarian and equipped with the knowledge and tools to meet the challenges they will encounter from Day One in practice. Support from the Australian Government Office of Learning and Teaching was used to address gaps in uniformity and currency in Australasian AWE teaching by developing the online OWP for shared teaching resources. The current report reviews the preceding individual articles on both students’ and educators’ rankings of various AWE topics. It allows veterinary educators to understand how the OWP and the resources within it were developed in response to the perceived needs of these two stakeholder groups. The current framework of the portal allows the addition of fresh material as these needs change and as international perspectives are embraced.

## Figures and Tables

**Table 1:** Online resources considered by the panel for use in the One Welfare learning and teaching portal (OWP) at the 2014 University of Sydney workshop.

Resource	Description	Comment
A negotiated curriculum.	Cohorts of students vote on topics they wish to learn in a given time period. This is an established method underpinned by literature (Brew and Barrie, 1999).	This method empowers and engages students, meaning they can focus on individual issues of interest and relevance and development of their own ethical frameworks. A pool of resources addressing each topic is required.
Scenarios for case-based learning.	Students access materials that require application of an ethical framework to practise addressing real life issues that they will encounter in practice. These materials can cover all aspects of professional practice.	Scenarios provide a safe but realistic learning experience, facilitating application of concepts, as well as learning by seeing how others do things.
The ‘human continuum.’	In the human continuum, students take a physical place in a line in response to a stimulus question, reflecting a continuum of perspectives on an issue. After peer discussion and negotiation, students reconsider their view on the position.	The human continuum engages students in debate, negotiation, reflective listening and peer learning. The development of this tool for online use would form a valuable resource for a variety of national and international collaborations and would promote valuable networking skills. This tool was further developed into ‘Chatterbox,’ the online tool designed to facilitate discussion of difficult and controversial ethical issues in a non-confrontational environment.
Quality of Life (QOL) Assessment Tool.	The QOL tool is primarily used as a teaching tool and is essentially an online calculator incorporating welfare scales from several sources. Students are asked to consider a variety of AWE issues and to estimate welfare impacts on animals.	This teaching tool encourages students to think deeply about AWE issues and form and defend a position stance. Estimation of the welfare impacts of disorders is innovative and informed by an impact score for dogs (28). The *doglogbook* app is one of the portal’s first steps in developing a QOL resource.
Team-based learning.	Team-based learning requires students to engage with course material prior to the class and then answer questions related to that material individually and in a group in order to identify knowledge gaps or misunderstandings. The class time is then spent on deep learning by applying knowledge to real life problems.	Team-based learning is a tool that can be used to both reinforce formal course content and develop communication and group work skills.
Personal reflection tools.	Several online questionnaires have been developed to investigate correlations between the attitudes of veterinary students towards animals. The OWP adapted one of these (24) and also further investigates impacts of demographic and experiential factors on attitudes towards animals as well students’ career aspirations.	Self-reflection is a valuable lifelong skill for veterinarians. Modified versions of the questionnaires could be used as a self-reflection tool for students. Online reflection tools can be used by students at the beginning and end of their use of the portal to gain understanding of their self-development.

**Table 2:** Topics considered according to seven areas of veterinary employment.

Area	Topics Proposed
General practice	The development of animal welfare science Reasons why animal welfare matters Science versus values (the merits of an evidence-based approach versus one’s own values in making decisions) Applied animal ethics (framework to guide ethical practice considering all stakeholders) Professional ethics (ethical responsibilities of a veterinarian)Laws and regulations regarding animal welfare Perspectives on welfare (e.g., international/trade, consumer, marketer, regulator) Human-animal bond (e.g., strength, emotional attachment). Triage (systematic protocol to establish urgency and severity and differentiate between emergencies and routine cases) Euthanasia
Production animals	Ethics of sustainable production (food security and animal welfare issues) Human-animal interactions and impacts on animalsIntensive vs extensive production systems Social, economic and cultural drivers of welfare outcomes Strategies to address painful husbandry procedures Distress associated with road, sea and air transportEuthanasia
Companion animals	Animal abuse/hoarding Behaviour and training Breeding Companion animal husbandry Cosmetic surgery NeuteringOver servicing Shelter medicine Euthanasia
Animals in the wild	Disaster preparedness Veterinarians’ duties to wild animals Methods and justification for their uses (e.g., harvesting/hunting, wildlife parks) Tensions between animal welfare and environmental concerns The nature and status of semi-owned animalsEuthanasia
Aquatic animals	Use of antibiotics Fishing (pain and distress associated with standard angling and trawling practices) Husbandry techniques (of farmed fish) Aquatic animal health and welfare issues Euthanasia
Animals kept for scientific purposes	3Rs (Replacement, Reduction and Refinement) AEC (Animal Ethics Committee) procedures or requirements Conscientious objections Humane endpoints What is a research animal? Euthanasia
Animals used in sport, recreation and display	Behaviour, selection and training for sport and recreation displays Educating the public Pushing of animals to physiological/behavioural limits Ownership/responsibility Euthanasia

**Table 3:** Panel of educators’ (*n* = 142) ranking of portal features.

Portal Feature	Ranking Median	Ranking Order
Scenarios and learning exercises	1	1
QOL assessment tool	3	2
A negotiated curriculum	4.5	3
The human continuum	4.5	3
Delivering welfare advice tool	5	5
Personal reflection tools	5.5	6
Automated search engines for hot topics	6.5	7
Team-based learning	6.5	7
Animal welfare science essays	8.5	9
A postgraduate suite	9	10

**Table 4:** Educators’ (*n* = 142) ranking of importance of Day One competences.

Topic	Ranked as Most Important	Ranked as Least Important
General practice	Professional ethics	Development of animal welfare science
	Euthanasia	
	Triage	
Production animals	Strategies to address painful husbandry procedures	Slaughter and preslaughter inspections
	Euthanasia	
	Ethics of sustainable production	
Companion animals	Euthanasia	Cosmetic surgery
	Companion animal husbandry	
	Neutering	
Animals in the wild	Veterinarian’s duties to wild animals	Methods and justification for their uses (hunting etc.)
	Euthanasia	
	Disaster preparedness	
Aquatic animals	Aquatic animal health and welfare issues	Euthanasia
	Husbandry techniques of farmed fish	
	Use of antibiotics	
Animals kept for scientific purposes	3Rs (Replacement, Reduction and Refinement)	Conscientious objections
	Humane end points	
	AEC procedures and requirements	
Animals used in sport, recreation and display	Ownership/responsibility	Educating the public
	Pushing animals to physiological/behavioural limits	
	Euthanasia	

**Table 5:** Summary reports on students’ opinions on welfare and ethics issues in various areas of veterinary practice and their importance among Day One competences.

Theme	Aim	Outcome	Report
Animals kept for scientific purposes	To determine the importance veterinary students place on their Day One competence in welfare and ethical decision-making regarding animals kept for scientific purposes.	Students ranked *AEC procedures or requirements* (median 2), the *3Rs* (median = 2) and *humane endpoints* (median = 2) as the three most important Day One competences, followed by *euthanasia, what is a research animal?* and *conscientious objections.* Female students ranked *conscientious objections*, *humane endpoints* and *euthanasia* higher than male students across all stages of study. A trend to score the latter topics higher as they progressed through the course was seen with male students while female students’ scores showed a trend to stay stable or decline. No differences with respect to gender were seen for *3Rs*, *AEC procedures or requirements* and *what is a research animal?*	Collins et al. (2018) [20]
Production animals	To examine the attitudes of veterinary science students to AWE issues in production animals.	Students ranked all nominated AWE issues in production animals of being at least moderate importance (mean score five or less). *Strategies to address painful husbandry procedures* (median = 2) was ranked as the most important topic for graduates, followed by the *ethics of sustainable production* (food security and animal welfare issues) (median = 2) with *differentiating between intensive and extensive animal systems in terms of their effects* (median = 4) ranking last. Stage of study influenced *human-animal interactions and associated impacts on production animals* with a decrease in ranking importance seen over the course of studies. Female students, regardless of stage of study, considered *distress associated with road, sea and air transport* to be more important than did male students. Female students also ranked *euthanasia* consistently higher at all stages of study compared to male students who ranked *euthanasia* lower in both early and late stages of study.	Cornish et al. (2018) [21]
Dogmanship and the *doglogbook* app	To use advancements in science and practice to help dog owners become more mindful of their dogs’ overall wellbeing. The problem of harm to humans resulting from dog behaviour issues or human misunderstanding of canine signals, and the problem of increased dog ownership pressures (urban living density, unfamiliarity with basic management requirements and time poor owners) results in decreased dog welfare.	Correct interpretations of canine body language and knowing how to behave around dogs are core skills for veterinarians in companion animal practice. They underpin safe human-dog interaction and are likely to reduce harm to humans and boost dog welfare. Good ‘dogmanship’ is defined as meeting dogs’ behavioural needs and allowing them to reach their full potential in a training setting and relies on the appropriate reading of, and response to, dog behaviour. The development of the science of dogmanship could be informed by progress in equitation science. Veterinarians are encouraged to become involved in, and encourage client use of, the *doglogbook* app which allows meticulous record of training and management activities and the sharing of this information with the appropriate professional.	McGreevy et al. (2017) [30]
Companion animals	To determine what veterinary students’ consider to be the most important AWE competences in companion animal practice on their first day of practice.	Students across all stages of study ranked *neutering* (median = 2), *companion animal husbandry* (median = 2), *euthanasia* (median = 2) *and behaviour and training* (median = 2) as the most important topics for Day One competence. More female students ranked AWE issues regarding *neutering* ‘extremely important’ to Day One competence than did male students. The topics that students ranked of ‘moderate’ importance were *animal abuse/hoarding*, *shelter medicine* and *animal breeding*, and stage of study influenced these rankings. The topics ranked as least important were *over servicing* (median = 6) and *cosmetic surgery* (median = 8) and no differences were seen in these responses due to gender or stage of study.	Degeling et al. (2017) [19]
Sport, recreation and display animals	To reveal what veterinary students consider to be important AWE topics for Day One Competence with respect to animals used in sport, recreation and display and to compare results with published data with respect to gender and stage of study.	Five topics were presented to students for consideration in the context of welfare of animals in sport, recreation and display. Students ranked *pushing animals to their physiologic/behavioural limits* (median = 2) as the most important concern. A greater number of female students ranked this as extremely important than did male students.	Fawcett et al. (2019) [22]
Wild animals	To determine what veterinary students, consider to be important for AWE competence when dealing with animals in the wild, and to determine correlations of these opinions with gender and stage of study.	The topics ranked most important were a *veterinarian’s duty to wild animals* (median = 1), followed by *euthanasia* (median = 2) and *disaster preparedness* (median = 3). The three topics ranked as least important were *methods and justifications for the use of wildlife* (median = 3), *the tension between welfare and environmental concerns* (median 3) and, last, *the status of semi-owned animals* (median 3). Students’ opinions on the importance of *euthanasia* differed by gender and stage of study–with importance increasing with stage of study and male students ranking it as more important than female students. Respondents considered *methods and justification for use* and *tensions between animal welfare concerns and environmental concerns* less important as their studies progressed.	Stafford et al. 2019 [23]
Aquatic animals	To determine what veterinary students consider to be important for AWE on their Day One competence when dealing with aquatic animals.	Students ranked all topics as being of high to moderate importance. Ranked most important were an *understanding of aquatic animals’ health and welfare issues* (median 2)*,* then *husbandry techniques of farmed fish* (median 2), *use of antibiotics* (median 3), *pain and distress associated with standard angling and trawling processes* (median 3), followed by *euthanasia* (median 3). Earlier stage students ranked the topic of *pain and distress associated with standard angling and trawling processes* higher than those in later stages of study. Female students initially rated *euthanasia* as more important than did male students, however, male students assigned *euthanasia* higher importance over time.	Lloyd et al. (2020) [24]
